# Supplementary material for: A systematic review of waterborne and water-related disease in animal populations of Florida from 1999–2019
Source: PLoS One. 2021 Jul 29;16(7):e0255025. doi: 10.1371/journal.pone.0255025 (PMC8321142; doi:10.1371/journal.pone.0255025)
Supplement: S2 File — (DOC) [file pone.0255025.s002.doc]

S2 File: Search Strings and Results by Database

| **Database** | **Search String** | **Results** |
| --- | --- | --- |
| ABI/INFORM Collection  Searched 4/2/20 | ab("waterborne disease" OR "water-based vector" OR "water-related toxin" OR "Amebic encephalitis" OR "Naegleria fowleri" OR "Primary amebic meningoencephalitis" OR arbovir* OR "California serogroup" OR "Jamestown Canyon" OR "Snowshoe Hare Virus" OR "La Crosse Virus" OR Campylobacter* OR Chickungunya OR Cholera OR "Vibrio cholerae" OR Ciguatera OR Cryptosporidi* OR Cyclospor* OR Dengue OR "Eastern Equine Encephalitis" OR "Escherichia coli" OR "Shiga Toxin" OR STEC OR Giardi* OR "Hepatitis A" OR "Hepatitis E" OR Legionell* OR Leptospir* OR Weil OR Malaria* OR Melioidosis OR Whitmore* OR "Burkholderia pseudomallei" OR "Neurotoxic Shellfish Poisoning" OR Brevetoxin* OR Polio* OR "Saint Louis Encephalitis" OR Salmonell* OR Saxitoxin OR "Paralytic Shellfish" OR Shigell* OR Tularemia OR "Typhoid" OR "Venezuelan Equine Encephalitis" OR Vibrio* OR "Viral Hemorrhagic" OR "Viral Haemorrhagic" OR "West Nile" OR "Yellow Fever" OR Zika) AND ab(Florid* OR "Southeastern United States")  **FILTERS:** Date: From January 01 1999 to December 31 2019; scholarly journals; English Only | 96 |
| Agricola  Searched 4/2/20 | Ab,ti(("waterborne disease" OR "water-based vector" OR "water-related toxin" OR "Amebic encephalitis" OR "Naegleria fowleri" OR "Primary amebic meningoencephalitis" OR arbovir* OR "California serogroup" OR "Jamestown Canyon" OR "Snowshoe Hare Virus" OR "La Crosse Virus" OR Campylobacter* OR Chickungunya OR Cholera OR "Vibrio cholerae" OR Ciguatera OR Cryptosporidi* OR Cyclospor* OR Dengue OR "Eastern Equine Encephalitis" OR "Escherichia coli" OR "Shiga Toxin" OR STEC OR Giardi* OR "Hepatitis A" OR "Hepatitis E" OR Legionell* OR Leptospir* OR Weil OR Malaria* OR Melioidosis OR Whitmore* OR "Burkholderia pseudomallei" OR "Neurotoxic Shellfish Poisoning" OR Brevetoxin* OR Polio* OR "Saint Louis Encephalitis" OR Salmonell* OR Saxitoxin OR "Paralytic Shellfish" OR Shigell* OR Tularemia OR "Typhoid" OR "Venezuelan Equine Encephalitis" OR Vibrio* OR "Viral Hemorrhagic" OR "Viral Haemorrhagic" OR "West Nile" OR "Yellow Fever" OR Zika) AND (Florid* OR "Southeastern United States"))  **FILTERS:** Date: From January 01 1999 to December 31 2019; scholarly journals; English Only | 30 |
| Agriculture Collection  Searched 4/8/20 | Keyword: "water borne" OR  Keyword: "waterborne diseases" OR  Keyword: water-based vector OR  Keyword: water-related toxin AND  Keyword: florid*OR  Keyword: "southeastern united states"  **FILTERS:** Date : Jan 01, 1999 - Dec 31, 2019 Sorted by: Academic Journals | 896 *(only 872 exported)* |
| Aquatic Sciences Collection  Searched 4/2/20 | AB,TI(("waterborne disease" OR "water-based vector" OR "water-related toxin" OR "Amebic encephalitis" OR "Naegleria fowleri" OR "Primary amebic meningoencephalitis" OR arbovir* OR "California serogroup" OR "Jamestown Canyon" OR "Snowshoe Hare Virus" OR "La Crosse Virus" OR Campylobacter* OR Chickungunya OR Cholera OR "Vibrio cholerae" OR Ciguatera OR Cryptosporidi* OR Cyclospor* OR Dengue OR "Eastern Equine Encephalitis" OR "Escherichia coli" OR "Shiga Toxin" OR STEC OR Giardi* OR "Hepatitis A" OR "Hepatitis E" OR Legionell* OR Leptospir* OR Weil OR Malaria* OR Melioidosis OR Whitmore* OR "Burkholderia pseudomallei" OR "Neurotoxic Shellfish Poisoning" OR Brevetoxin* OR Polio* OR "Saint Louis Encephalitis" OR Salmonell* OR Saxitoxin OR "Paralytic Shellfish" OR Shigell* OR Tularemia OR "Typhoid" OR "Venezuelan Equine Encephalitis" OR Vibrio* OR "Viral Hemorrhagic" OR "Viral Haemorrhagic" OR "West Nile" OR "Yellow Fever" OR Zika) AND (Florid* OR "Southeastern United States"))  **FILTERS:** Date: From January 01 1999 to December 31 2019; scholarly journals only; English only | 213 |
| Biological Sciences  Searched 4/8/20 | **AB,TI**(("waterborne disease" OR "water-based vector" OR "water-related toxin" OR "Amebic encephalitis" OR "Naegleria fowleri" OR "Primary amebic meningoencephalitis" OR arbovir* OR "California serogroup" OR "Jamestown Canyon" OR "Snowshoe Hare Virus" OR "La Crosse Virus" OR Campylobacter* OR Chickungunya OR Cholera OR "Vibrio cholerae" OR Ciguatera OR Cryptosporidi* OR Cyclospor* OR Dengue OR "Eastern Equine Encephalitis" OR "Escherichia coli" OR "Shiga Toxin" OR STEC OR Giardi* OR "Hepatitis A" OR "Hepatitis E" OR Legionell* OR Leptospir* OR Weil OR Malaria* OR Melioidosis OR Whitmore* OR "Burkholderia pseudomallei" OR "Neurotoxic Shellfish Poisoning" OR Brevetoxin* OR Polio* OR "Saint Louis Encephalitis" OR Salmonell* OR Saxitoxin OR "Paralytic Shellfish" OR Shigell* OR Tularemia OR "Typhoid" OR "Venezuelan Equine Encephalitis" OR Vibrio* OR "Viral Hemorrhagic" OR "Viral Haemorrhagic" OR "West Nile" OR "Yellow Fever" OR Zika) AND (Florid* OR "Southeastern United States"))  **FILTERS:** Date: From January 01 1999 to December 31 2019; scholarly journals only; English | 1,292 |
| BioOne Complete  Searched 4/2/20 | ABSTRACT:([[“waterborne disease”] OR [“water-based vector”] OR [“water-related toxin”] OR [“Amebic encephalitis”] OR [“Naegleria fowleri”] OR [“Primary amebic meningoencephalitis”] OR [arbovir*] OR [“California serogroup”] OR [“Jamestown Canyon”] OR [“Snowshoe Hare Virus”] OR [“La Crosse Virus”] OR [Campylobacter] OR [Chickungunya] OR [Cholera] OR [“Vibrio cholerae”] OR [Ciguatera] OR [Cryptosporidi*] OR [Cyclospor*] OR [Dengue] OR [“Eastern Equine Encephalitis”] OR [“Escherichia coli”] OR [“Shiga Toxin”] OR [STEC] OR [Giardi*] OR [“Hepatitis A”] OR [“Hepatitis E”] OR [Legionell*] OR [Leptospir*] OR [Weil] OR [Malaria*] OR [Melioidosis] OR [Whitmore*] OR [“Burkholderia pseudomallei”] OR [“Neurotoxic Shellfish Poisoning”] OR [Brevetoxin*] OR [Polio*] OR [“Saint Louis Encephalitis”] OR [Salmonell*] OR [Saxitoxin] OR [“Paralytic Shellfish”] OR [Shigell*] OR [Tularemia] OR [Typhoid] OR [“Venezuelan Equine Encephalitis”] OR [Vibrio*] OR [“Viral Hemorrhagic”] OR [“Viral Haemorrhagic”] OR [“West Nile”] OR [“Yellow Fever”] OR [Zika]] )  **FILTERS:** Date: 1999-2019 (*No location in first search string*) | 77 |
| Environment Complete  Searched 4/2/20 | AB((waterborne disease OR water-based vector OR water-related toxin) AND (Amebic encephalitis OR Naegleria fowleri OR Primary amebic meningoencephalitis OR arbovir* OR California serogroup OR Jamestown Canyon OR Snowshoe Hare Virus OR “La Crosse Virus” OR Campylobacter OR Chickungunya OR Cholera OR Vibrio cholerae OR Ciguatera OR Cryptosporidi* OR Cyclospor* OR Dengue OR Eastern Equine Encephalitis OR Escherichia coli OR Shiga Toxin OR STEC OR Giardi* OR Hepatitis A OR Hepatitis E OR Legionell* OR Leptospir* OR Weil OR Malaria* OR Melioidosis OR Whitmore* OR Burkholderia pseudomallei OR Neurotoxic Shellfish Poisoning OR Brevetoxin* OR Polio* OR Saint Louis Encephalitis OR Salmonell* OR Saxitoxin OR Paralytic Shellfish OR Shigell* OR Tularemia OR Typhoid OR Venezuelan Equine Encephalitis OR Vibrio* OR Viral Hemorrhagic OR Viral Haemorrhagic OR West Nile OR Yellow Fever OR Zika) AND (Florid* OR “Southeastern United States”))  **FILTERS:** Date: From January 1999 to December 2019; academic journals only; | 597 |
| Agricultural and Environmental Science Collection  Searched 4/8/20 | **AB,TI**((waterborne disease OR water-based vector OR water-related toxin) AND (Amebic encephalitis OR Naegleria fowleri OR Primary amebic meningoencephalitis OR arbovir* OR California serogroup OR Jamestown Canyon OR Snowshoe Hare Virus OR "La Crosse Virus" OR Campylobacter OR Chickungunya OR Cholera OR Vibrio cholerae OR Ciguatera OR Cryptosporidi* OR Cyclospor* OR Dengue OR Eastern Equine Encephalitis OR Escherichia coli OR Shiga Toxin OR STEC OR Giardi* OR "Hepatitis A" OR "Hepatitis E" OR Legionell* OR Leptospir* OR Weil OR Malaria* OR Melioidosis OR Whitmore* OR Burkholderia pseudomallei OR Neurotoxic Shellfish Poisoning OR Brevetoxin* OR Polio* OR "Saint Louis Encephalitis" OR Salmonell* OR Saxitoxin OR Paralytic Shellfish OR Shigell* OR Tularemia OR Typhoid OR "Venezuelan Equine Encephalitis" OR Vibrio* OR Viral Hemorrhagic OR Viral Haemorrhagic OR West Nile OR Yellow Fever OR Zika) AND (Florid* OR "Southeastern United States"))  **FILTERS:** Date: From January 01 1999 to December 31 2019; scholarly journals only; English only | 1 |
| Environmental Studies and Policy  Searched 4/8/20 | Keyword: "water borne" OR  Keyword: "waterborne diseases" OR  Keyword: “water-based vector” OR  Keyword: “water-related toxin” AND  Keyword: “Florida” OR  Keyword: “floridian” OR  Keyword: "southeastern united states"  **FILTERS:** Peer-Reviewed Journal: Document type= Article; Date : Jan 01, 1999 - Dec 31, 2019 | 477 |
| Google Scholar  Searched 4/8/20 | all abstract: ("waterborne disease" OR "water-based vector" OR "water-related toxin") AND (Florida OR Floridian OR “Southeastern United States”)  **FILTERS:** Date range: 1999-2019; Filter- do not include citations; do not include patents; English Pages only | 1,930  *(only 1,280 exported)* |
| Health and Medicine  Searched 4/3/20 | ab(((waterborne disease OR water-based vector OR water-related toxin) AND (Amebic encephalitis OR Naegleria fowler OR Primary amebic meningoencephalitis OR arbovir* OR California serogroup OR Jamestown Canyon OR Snowshoe Hare Virus OR La Crosse Virus OR Campylobacter OR Chickungunya OR Cholera OR Vibrio cholera OR Ciguatera OR Cryptosporidi* OR Cyclospor* OR Dengue OR Eastern Equine Encephalitis OR Escherichia coli OR Shiga Toxin OR STEC OR Giardi* OR Hepatitis A OR Hepatitis E OR Legionell* OR Leptospir* OR Weil OR Malaria* OR Melioidosis OR Whitmore* OR Burkholderia pseudomallei OR Neurotoxic Shellfish Poisoning OR Brevetoxin* OR Polio* OR Saint Louis Encephalitis OR Salmonell* OR Saxitoxin OR Paralytic Shellfish OR Shigell* OR Tularemia OR Typhoid OR Venezuelan Equine Encephalitis OR Vibrio* OR Viral Hemorrhagic OR Viral hemorrhagic OR West Nile OR Yellow Fever OR Zika) AND (Florid* OR "Southeastern United States")))  **FILTERS:** Date: From January 01 1999 to December 31 2019; English only | 1 |
| MEDLINE  Searched 4/3/20 | ab(((waterborne disease OR water-based vector OR water-related toxin) AND (Amebic encephalitis OR Naegleria fowler OR Primary amebic meningoencephalitis OR arbovir* OR California serogroup OR Jamestown Canyon OR Snowshoe Hare Virus OR La Crosse Virus OR Campylobacter OR Chickungunya OR Cholera OR Vibrio cholera OR Ciguatera OR Cryptosporidi* OR Cyclospor* OR Dengue OR Eastern Equine Encephalitis OR Escherichia coli OR Shiga Toxin OR STEC OR Giardi* OR Hepatitis A OR Hepatitis E OR Legionell* OR Leptospir* OR Weil OR Malaria* OR Melioidosis OR Whitmore* OR Burkholderia pseudomallei OR Neurotoxic Shellfish Poisoning OR Brevetoxin* OR Polio* OR Saint Louis Encephalitis OR Salmonell* OR Saxitoxin OR Paralytic Shellfish OR Shigell* OR Tularemia OR Typhoid OR Venezuelan Equine Encephalitis OR Vibrio* OR Viral Hemorrhagic OR Viral hemorrhagic OR West Nile OR Yellow Fever OR Zika) AND (Florid* OR "Southeastern United States")))  **FILTERS:** Date: From January 01 1999 to December 31 2019 | 0 |
| Nursing and Allied Health Outcomes  Searched 4/8/20 | Keyword: "water borne" OR  Keyword: "waterborne diseases" OR  Keyword: “water-based vector” OR  Keyword: “water-related toxin” AND  Keyword: “florida” OR  Keyword: “floridian” OR  Keyword: "southeastern united states"  **FILTERS:** Peer-Reviewed; Date : Jan 01, 1999 - Dec 31, 2019 | 830  (only 812 exported) |
| Pubmed  Searched 4/2/20 | ((((“waterborne disease” [tiab] OR “water-based vector” [tiab] OR “water-related toxin” [tiab] OR "Amebic encephalitis" [tiab] OR “Naegleria fowleri” [tiab] OR “Primary amebic meningoencephalitis” [tiab] OR arbovir* [tiab] OR “California serogroup” [tiab] OR “Jamestown Canyon” [tiab] OR “Snowshoe Hare Virus” [tiab] OR “La Crosse Virus” [tiab] OR Campylobacter* [tiab] OR Chickungunya [tiab] OR Cholera [tiab] OR “Vibrio cholerae” [tiab] OR Ciguatera [tiab] OR Cryptosporidi* [tiab] OR Cyclospor* [tiab] OR Dengue [tiab] OR “Eastern Equine Encephalitis” [tiab] OR “Escherichia coli” [tiab] OR “Shiga Toxin” [tiab] OR STEC [tiab] OR Giardi* [tiab] OR “Hepatitis A” [tiab] OR “Hepatitis E” [tiab] OR Legionell* [tiab] OR Leptospir* [tiab] OR Weil [tiab] OR Malaria* [tiab] OR Melioidosis [tiab] OR Whitmore* [tiab] OR “Burkholderia pseudomallei” [tiab] OR “Neurotoxic Shellfish Poisoning” [tiab] OR Brevetoxin* [tiab] OR Polio* [tiab] OR “Saint Louis Encephalitis” [tiab] OR Salmonell* [tiab] OR Saxitoxin [tiab] OR “Paralytic Shellfish” [tiab] OR Shigell* [tiab] OR Tularemia [tiab] OR “Typhoid” [tiab] OR “Venezuelan Equine Encephalitis” [tiab] OR Vibrio* [tiab] OR “Viral Hemorrhagic” [tiab] OR “Viral Haemorrhagic” [tiab] OR “West Nile” [tiab] OR “Yellow Fever” [tiab] OR Zika [tiab]) AND (Florid* [tiab] OR “Southeastern United States” [tiab]))) AND ( "1999/01/01"[PDat] : "2019/12/31"[PDat] ))  **FILTERS:** English; search sting includes date range | 684 |
| SAGE Journals  Searched 4/2/20 | Search abstracts for these keywords:  (("waterborne disease" OR "water-based vector" OR "water-related toxin" OR "Amebic encephalitis" OR "Naegleria fowleri" OR "Primary amebic meningoencephalitis" OR arbovir* OR "California serogroup" OR "Jamestown Canyon" OR "Snowshoe Hare Virus" OR "La Crosse Virus" OR Campylobacter* OR Chickungunya OR Cholera OR "Vibrio cholerae" OR Ciguatera OR Cryptosporidi* OR Cyclospor* OR Dengue OR "Eastern Equine Encephalitis" OR "Escherichia coli" OR "Shiga Toxin" OR STEC OR Giardi* OR "Hepatitis A" OR "Hepatitis E" OR Legionell* OR Leptospir* OR Weil OR Malaria* OR Melioidosis OR Whitmore* OR "Burkholderia pseudomallei" OR "Neurotoxic Shellfish Poisoning" OR Brevetoxin* OR Polio* OR "Saint Louis Encephalitis" OR Salmonell* OR Saxitoxin OR "Paralytic Shellfish" OR Shigell* OR Tularemia OR "Typhoid" OR "Venezuelan Equine Encephalitis" OR Vibrio* OR "Viral Hemorrhagic" OR "Viral Haemorrhagic" OR "West Nile" OR "Yellow Fever" OR Zika) AND (Florid* OR "Southeastern United States"))  **FILTERS:** Date Range: 1999-2019 | 169 |
| Science Direct  Searched 4/2/20 | Find articles with these terms: (Florida OR Floridian OR "Southeastern United States")  Title, abstract, and keywords: ("water borne" OR "waterborne disease" OR "water-based vector" OR "water-related toxin")  **FILTERS:** Date restrictions: 1999-2019; Article Types: Review Articles, Research Articles, Case Reports, Data Articles, Mini Reviews, Replication Studies | 33 |
| SpringerLink Journals  Searched 4/8/20 | ("waterborne disease" OR "water-based vector" OR "water-related toxin" OR "Amebic encephalitis" OR "Naegleria fowleri" OR "Primary amebic meningoencephalitis" OR arbovir* OR "California serogroup" OR "Jamestown Canyon" OR "Snowshoe Hare Virus" OR "La Crosse Virus" OR Campylobacter* OR Chickungunya OR Cholera OR "Vibrio OR cholerae" OR Ciguatera OR Cryptosporidi* OR Cyclospor* OR Dengue OR "Eastern Equine Encephalitis" OR "Escherichia coli" OR "Shiga Toxin" OR STEC OR Giardi* OR "Hepatitis A" OR "Hepatitis E" OR Legionell* OR Leptospir* OR Weil OR Malaria* OR Melioidosis OR Whitmore* OR "Burkholderia pseudomallei" OR "Neurotoxic Shellfish Poisoning" OR Brevetoxin* OR Polio* OR "Saint Louis Encephalitis" OR Salmonell* OR Saxitoxin OR "Paralytic Shellfish" OR Shigell* OR Tularemia OR "Typhoid" OR "Venezuelan Equine Encephalitis" OR Vibrio* OR "Viral Hemorrhagic" OR "Viral Haemorrhagic" OR "West Nile" OR "Yellow Fever" OR Zika) AND ((Florid* OR "Southeastern United States")'  **FILTERS:** English only, article only, and between 1999-2019 | 413 |
| TOXLINE  Searched 4/2/20 | AB(("waterborne disease" OR "water-based vector" OR "water-related toxin" OR "Amebic encephalitis" OR "Naegleria fowleri" OR "Primary amebic meningoencephalitis" OR arbovir* OR "California serogroup" OR "Jamestown Canyon" OR "Snowshoe Hare Virus" OR "La Crosse Virus" OR Campylobacter* OR Chickungunya OR Cholera OR "Vibrio cholerae" OR Ciguatera OR Cryptosporidi* OR Cyclospor* OR Dengue OR "Eastern Equine Encephalitis" OR "Escherichia coli" OR "Shiga Toxin" OR STEC OR Giardi* OR "Hepatitis A" OR "Hepatitis E" OR Legionell* OR Leptospir* OR Weil OR Malaria* OR Melioidosis OR Whitmore* OR "Burkholderia pseudomallei" OR "Neurotoxic Shellfish Poisoning" OR Brevetoxin* OR Polio* OR "Saint Louis Encephalitis" OR Salmonell* OR Saxitoxin OR "Paralytic Shellfish" OR Shigell* OR Tularemia OR "Typhoid" OR "Venezuelan Equine Encephalitis" OR Vibrio* OR "Viral Hemorrhagic" OR "Viral Haemorrhagic" OR "West Nile" OR "Yellow Fever" OR Zika) AND (Florid* OR "Southeastern United States"))  **FILTERS:** Date: From January 01 1999 to December 31 2019; scholarly journals only | 112 |
| Web of Science Core Collection  Searched 4/2/20 | TS= (("waterborne disease" OR "water-based vector" OR "water-related toxin" OR "Amebic encephalitis" OR "Naegleria fowleri" OR "Primary amebic meningoencephalitis" OR arbovir* OR "California serogroup" OR "Jamestown Canyon" OR "Snowshoe Hare Virus" OR "La Crosse Virus" OR Campylobacter* OR Chickungunya OR Cholera OR "Vibrio cholerae" OR Ciguatera OR Cryptosporidi* OR Cyclospor* OR Dengue OR "Eastern Equine Encephalitis" OR "Escherichia coli" OR "Shiga Toxin" OR STEC OR Giardi* OR "Hepatitis A" OR "Hepatitis E" OR Legionell* OR Leptospir* OR Weil OR Malaria* OR Melioidosis OR Whitmore* OR "Burkholderia pseudomallei" OR "Neurotoxic Shellfish Poisoning" OR Brevetoxin* OR Polio* OR "Saint Louis Encephalitis" OR Salmonell* OR Saxitoxin OR "Paralytic Shellfish" OR Shigell* OR Tularemia OR "Typhoid" OR "Venezuelan Equine Encephalitis" OR Vibrio* OR "Viral Hemorrhagic" OR "Viral Haemorrhagic" OR "West Nile" OR "Yellow Fever" OR Zika) AND (Florid* OR "Southeastern United States"))  **FILTERS:** Timespan=1999-2019; Article or Review; English only | 1,204 |
|  | **Total** | **8,363** |
