# Supplementary material for: A systematic review of waterborne and water-related disease in animal populations of Florida from 1999–2019
Source: PLoS One. 2021 Jul 29;16(7):e0255025. doi: 10.1371/journal.pone.0255025 (PMC8321142; doi:10.1371/journal.pone.0255025)
Supplement: Attachment — Submitted filename: PONE animal sys rev.docx [file pone.0255025.s003.docx]

Reviewer summary

The study entitled “A Systematic Review of Waterborne and Water-Related Disease in Animal Populations of Florida from 1999-2019” provides a summary of reports of animal diseases with public health relevance in Florida based on a systematic review of primary scientific literature. This study is timely as animals are increasingly recognized as an important reservoir for human disease agents.

The authors report on the numbers of studies associated with various types of animals, various types of disease agents, and locations (counties) in Florida. However, they do not report or analyze any quantitative data from the studies of interest, and are thus unable to draw conclusions about the risk or prevalence of various animal-associated human disease agents in Florida. It is unclear if or how the number of papers reporting on a given animal-associated disease agent correlates with meaningful information about animal-associated disease risks in the state. Despite this, it may be useful for vets, health officials, policy makers, and scientists to have this summary of research done on various animals and disease agents in Florida. To this end, the paper would benefit from a map, table and/or figure summarizing included studies by animal type, disease agent, and location.

Synthesizing the information from their review would also help the authors meet PLOS One’s criteria for systematic reviews, which states that the journal accepts systematic reviews that use “explicit, systematic methods to identify, select, and critically appraise relevant research, and to collect and analyze data from the studies that are included in the review”. While the authors here systematically identified the studies they have summarized, they did not present new ideas, analyses, or conclusions based on the published data. This manuscript, particularly the discussion, reads more as a traditional review.

Throughout the manuscript, the authors should clarify and differentiate the different types of disease agents they’re interested in. There are many instances where toxins are grouped in with zoonotic pathogens, and these are very different types of public health risks. Specific instances of where toxins and pathogens should be differentiated are indicated below in the specific comments. In addition, it is not clear from the text how various types of organisms are associated with animals and humans. For example, K. brevis is not itself a pathogen or toxin, but it produces toxins that bioaccumulate in seafood and is dangerous to humans when ingested. The various types of disease agents and links between human illness, animals, and the environment should perhaps be better described.

Though not grammatically or factually incorrect, some of the text is written in a style that is not typical of scientific manuscripts. Several specific examples where extra words or descriptors are used and could be removed are noted below, and the study authors should work with the journal’s editors to remove unnecessary text prior to publication.

Specific comments

Abstract

- Lines 26-27: The term “infectious” is implied for pathogens and confusing here because the authors could also be referring to toxins (which are not infectious), suggest removing.
- Lines 27-28: Diseases/illnesses in Florida are reportable to the department of health regardless of whether they’re occurring in residents or in visitors to the state, correct? Suggest rewording to clarify.
- Line 29: Suggest authors amend text to “water-related pathogens and toxins” here, since both toxins and pathogens are discussed.
- Lines 28-30: Suggest that the authors update text to reflect that the systematic review also aimed to understand to what degree various water-related pathogens are associated with various animal types and their risk to public health.
- Suggest including which waterborne diseases and which animals were included in the searches in the abstract; this is covered later in the manuscript but is central to the study’s aims and methods and should be briefly described in the abstract.
- Line 36: What is meant by “original data”? (Presence/absence? Abundance? By any method?)
- Line 38: Large numbers should not be written as text (70 v. seventy).

Introduction

- Lines 57-28: Mention of them eparks seems irrelevant to discussion of Florida’s waters; suggest removing.
- Line 60: It’s distracting and unnecessary to specify that Florida’s residents are humans.
- Line 62: Most of these animals are not “Florida-specific” because they are also found in other areas, suggest removing.
- Paragraph at line 72: Suggest introducing waterborne infectious diseases, water-related vector-borne diseases, and toxins as related but separate categories of disease agents before defining them. In general, authors should be sure that toxins in particular are differentiated from infectious disease agents. It would also be helpful to have examples of each of these disease agent categories explaining animal association link to human disease.
- Line 76: Fix typo “are can be”.
- Line 78: Toxin-producing organisms are not themselves “contaminated” organisms, perhaps the authors meant to that they “contaminate” water bodies? Please clarify.
- Line 86: Run-on sentence, suggest splitting into at least two sentences.
- Line 91: Replace “have been impacting” with “impact”.

Methods

- Paragraph beginning at line 97 and Table 1: Are all of these water-related diseases related to animals? It’s not clear from the text or table legend. Based on the list of disease agents, this appears to be the case, but the authors should clarify. It would also be useful to include information on what types of animals these various diseases are associated with.
- Line 107: Suggest providing a short description of PRISMA guidelines.
- Line 138: What types of original data were included? Suggest providing details and/or examples.
- Lines 145 and 149: Not necessary to specify that articles were screened or reviewed by the coauthors.
- Lines 148-149: Item “d” specifies pathogens, but does this include toxins? Please clarify.
- Line 158-162: Syntax and organization of list is distracting, please re-word.
- Consider moving Figure 1 to methods section.

Results

- Figure 1: Consider specifying on figure or in legend why records were excluded during screening; information is included in the text but would be helpful in the figure.
- Line 175: Suggest updating paragraph header to reflect the fact that both pathogens and toxins are discussed.
- Line 191: *K. brevis* isn’t a pathogen, it’s a toxin-producing organism. Please clarify.
- Suggest including a map with colors to indicate where in the state the authors are reporting studies on various types of animal disease and what types of pathogens are there. This could help vets and public health officials understand where animal diseases of public health relevance have been reported.
- Suggest creating a table or figure with results broadly summarized/synthesized, reflecting text; for example, it would be helpful to see how many studies were associated with each disease agent, animal type, and location, rather than a list of studies.

Discussion

- Line 231: Add period after citations.
- Line 296: K. brevis isn’t a toxin, it produces toxins (brevetoxins).
- Line 312: Toxins are not pathogens, please revise text.
- In general, the discussion is focused on recommendations to reduce risks associated with different types of animal-associated human disease agents, and broad descriptions of included study results. However, the systematic review was aimed at enumerating reports of animal-associated human disease agents, and it is not clear how the results of the review inform the key points in the discussion. Suggest that the authors revise the discussion with a focus on describing broad trends (animals, diseases, and locations) they found in their literature search, how this information could be utilized by the state and/or vets and public health personnel, and identifying opportunities for additional research.
