# Supplementary material for: A systematic review of waterborne and water-related disease in animal populations of Florida from 1999–2019
Source: PLoS One. 2021 Jul 29;16(7):e0255025. doi: 10.1371/journal.pone.0255025 (PMC8321142; doi:10.1371/journal.pone.0255025)
Supplement: Attachment — Submitted filename: PLOS_ONE_ReviewerResponse.docx [file pone.0255025.s004.docx]

**Response to Comments from Reviewers**

The authors would like to thank the reviewers for highlighting areas of weakness in our original submission. We believe in the importance of this study and the publication of its findings. By providing such valuable feedback, the authors were able to work together to reassess the findings and reread the studies to hopefully prepare a stronger manuscript. We are willing to continue shaping and revising our study findings as needed to adequately present our work to the reviewers and to a larger audience. We are very appreciative for the opportunity to resubmit and we appreciate your feedback.

**Response to Reviewers**

| **Overall Manuscript Comments/Requests** | **Reply/Revision** |
| --- | --- |
| The authors report on the numbers of studies associated with various types of animals, various types of disease agents, and locations (counties) in Florida. However, they do not report or analyze any quantitative data from the studies of interest, and are thus unable to draw conclusions about the risk or prevalence of various animal-associated human disease agents in Florida. It is unclear if or how the number of papers reporting on a given animal-associated disease agent correlates with meaningful information about animal-associated disease risks in the state. Despite this, it may be useful for vets, health officials, policy makers, and scientists to have this summary of research done on various animals and disease agents in Florida. To this end, the paper would benefit from a map, table and/or figure summarizing included studies by animal type, disease agent, and location. | - The authors appreciate this guidance by the reviewers and have thoroughly reviewed and reassessed the included articles for quantitative data. This has resulted in an updated table. Per the reviewer’s suggestion, additional information was added for prevalence data per animals sampled, when available. A significant challenge to doing any further analysis on the collective results, particularly as they relate to disease risk, is the wide-ranging styles and formats in the study results and findings from each paper, particularly in comparison across discipline and time. Many papers that appear to have been written for a biological or zoological audience do not include prevalence for the number of animals samples but instead for the number of specimens tested. Often animals in the studies provided multiple samples and it was difficult to ascertain the disease risk from the data presented. A further recommendation has been added to the discussion section to consider a wider-ranging audience, particularly those in public health, when the biological sciences are reporting their animal-specific findings. This would ideally allow for better use of the published data for these pathogens and toxins of public and veterinary importance. - A new map figure has been created to summarize the included studies by animal type, location and disease agent as recommended by the reviewer (See Figure 2). |
| Synthesizing the information from their review would also help the authors meet PLOS One’s criteria for systematic reviews, which states that the journal accepts systematic reviews that use “explicit, systematic methods to identify, select, and critically appraise relevant research, and to collect and analyze data from the studies that are included in the review”. While the authors here systematically identified the studies they have summarized, they did not present new ideas, analyses, or conclusions based on the published data. This manuscript, particularly the discussion, reads more as a traditional review. | - The authors recognize the feedback provided by the reviewers but still feel the manuscript offers a novel assessment of the information from the included studies as they pertain to human health and zoonotic disease transmission. While analysis of the multiple disease and toxins studies, as well as the multiple laboratory methods, animal species, specimen types, and reporting formats would have provided a richer examination of the overall disease risk for humans and animals, due to the varied styles and study designs, it was out of the scope of this current review. We hope that our review will provide a look into the pathogens and toxins that have been circulating simultaneously along the cases reported in Florida residents and visitors and will spur new research endeavors by One Health teams across Florida’s human, animal, and environmental health sectors. |
| Throughout the manuscript, the authors should clarify and differentiate the different types of disease agents they’re interested in. There are many instances where toxins are grouped in with zoonotic pathogens, and these are very different types of public health risks. Specific instances of where toxins and pathogens should be differentiated are indicated below in the specific comments. In addition, it is not clear from the text how various types of organisms are associated with animals and humans. For example, K. brevis is not itself a pathogen or toxin, but it produces toxins that bioaccumulate in seafood and is dangerous to humans when ingested. The various types of disease agents and links between human illness, animals, and the environment should perhaps be better described. | - The authors are grateful for this revision note and the manuscript has been edited to include more disease agent differentiation and language to indicate pathogen v. toxin throughout. - All pathogens and/or toxins were selected as they are reportable to the Florida Department of Health and most have a known zoonotic transmission potential. The discussion section attempted to outline the most common ways in which humans and/or animals may be exposed to these agents (ex. *K. brevis* and other toxins in lines 369-382 of the resubmitted tracked document). But, due to brevity and the amount of agents examined, the authors found it difficult to detail extensive transmission routes and pathways for all pathogens or toxins in humans and animals. However it could be a beneficial follow up study to review the exposure risks for the agents found in this paper for Florida’s animals and humans. |
| Though not grammatically or factually incorrect, some of the text is written in a style that is not typical of scientific manuscripts. Several specific examples where extra words or descriptors are used and could be removed are noted below, and the study authors should work with the journal’s editors to remove unnecessary text prior to publication. | - The authors have provided additional edits as advised by the reviewers but additional revisions can be facilitated to adhere to a preferred writing style for the journal or reviewers. |
| The discussion included both strengths and limitations of the study design and presented the authors’ conclusion as to best handle the specific areas which are lacking. The authors such a OneHealth approach to help populate missing links in an interdisciplinary manner. The reviewer finds this approach interesting and this is the one aspect of the manuscript which lacks more information, but it also may be beyond the scope of the authors’ original intent. | - The authors have added additional information to the discussion per the advice of the reviewers. While this is an area of the study that is perhaps deserving of its own manuscript, the authors have tried to hit on the major recommendations to implementing a One Health approach while paying attention to the need for brevity. However, if the reviewers would like, we would be happy to add more to this section of the manuscript as we also feel it is important. |
| **Specific Manuscript Comments/Requests** | **Reply/Revision- Meg and Sabrina** |
| **Abstract**   - Lines 26-27: The term “infectious” is implied for pathogens and confusing here because the authors could also be referring to toxins (which are not infectious), suggest removing. | - Removed the term “infectious” as advised. |
| - Lines 27-28: Diseases/illnesses in Florida are reportable to the department of health regardless of whether they’re occurring in residents or in visitors to the state, correct? Suggest rewording to clarify. | - Changed the term “residents” to “people” to be inclusive of anyone in the state, regardless of state residency, for clarification. |
| - Line 29: Suggest authors amend text to “water-related pathogens and toxins” here, since both toxins and pathogens are discussed. | - Inserted the phrase “and toxins' both here and throughout the manuscript. |
| - Lines 28-30: Suggest that the authors update text to reflect that the systematic review also aimed to understand to what degree various water-related pathogens are associated with various animal types and their risk to public health. | - The authors would have liked this opportunity but feel that investigating a specific risk association is outside the scope of this paper as some of the earlier studies in this analysis did not indicate prevalence data. Language was added to the limitations of the paper (page 29 of the resubmitted tracked document). This is an excellent recommendation for further research. |
| - Suggest including which waterborne diseases and which animals were included in the searches in the abstract; this is covered later in the manuscript but is central to the study’s aims and methods and should be briefly described in the abstract. | - While the authors agree that this information would be helpful in the abstract, the list of pathogens and toxins used in the search would put the abstract well beyond the 300 word count for the journal. Several agents and resulting disease were highlighted in the results section of the abstract and Table 1 describes each condition of interest for this study. |
| - Line 36: What is meant by “original data”? (Presence/absence? Abundance? By any method?) | - Changed the term “original data” to “primary research findings” to clarify that only primary studies were included and secondary data analysis or reviews were excluded. |
| - Line 38: Large numbers should not be written as text (70 v. seventy). | - Changed the term “seventy” to the numeric “70” as suggested. |
| **Introduction**   - Lines 57-28: Mention of theme parks seems irrelevant to discussion of Florida’s waters; suggest removing. | - The sentence related to theme parks has been removed. |
| - Line 60: It’s distracting and unnecessary to specify that Florida’s residents are humans. | - This sentence has been revised as suggested to remove the unnecessary wording for ‘humans’ and tourists |
| - Line 62: Most of these animals are not “Florida-specific” because they are also found in other areas, suggest removing. | - The term “Florida-specific” has been removed |
| - Paragraph at line 72: Suggest introducing waterborne infectious diseases, water-related vector-borne diseases, and toxins as related but separate categories of disease agents before defining them. In general, authors should be sure that toxins in particular are differentiated from infectious disease agents. It would also be helpful to have examples of each of these disease agent categories explaining animal association link to human disease. | - The authors appreciate this suggestion and added the following language, *“Waterborne infectious diseases are caused by pathogenic microbes that reside in water and can cause negative symptoms in both humans and animals if ingested. Water-related vector-borne diseases are also caused by pathogenic microbes but require a vector for transmission to humans. Finally, water-related toxins are harmful substances which are naturally produced by organisms that reside in the water.”* |
| - Line 76: Fix typo “are can be”. | - The grammatical error has been fixed |
| - Line 78: Toxin-producing organisms are not themselves “contaminated” organisms, perhaps the authors meant to that they “contaminate” water bodies? Please clarify. | - The wording for this sentence has been revised as suggested by reviewers to read, “*Water-based toxins are infections that arise from contact with an organism that resides in water and has the ability to contaminate water bodies*” |
| - Line 86: Run-on sentence, suggest splitting into at least two sentences. | - This run-on sentence has been fixed to read, “*Yet, these reportable water-related diseases vary widely in their exposure risks, their transmission routes, and their disease presentations. These factors make it difficult to coordinate surveillance efforts between state public health and veterinary professionals or develop a universally-applicable approach to decrease their burden.”* |
| - Line 91: Replace “have been impacting” with “impact”. | - Original text “*Have been impacting*” has been replaced with “*impact*” |
| **Methods**   - Paragraph beginning at line 97 and Table 1: Are all of these water-related diseases related to animals? It’s not clear from the text or table legend. Based on the list of disease agents, this appears to be the case, but the authors should clarify. It would also be useful to include information on what types of animals these various diseases are associated with. | - The authors recognize the confusion indicated by the reviewer feedback. As part of the purpose of this study was to see which of these water-related diseases that are reportable in Florida have also been found in animal populations within the state, additional language has been added to indicate why these diseases are investigated within this study - In addiiton, a footnote was added to Table 1 to indicate that Poliomyelitis does not have a known animal reservoir |
| - Line 107: Suggest providing a short description of PRISMA guidelines. | - Additional language was added to describe the PRIMSA provisions and process as prompted by the reviewer |
| - Line 138: What types of original data were included? Suggest providing details and/or examples. | - As advised, the authors revised and expanded on the language describing the inclusion criteria for primary study findings and provided example of what primary findings would constitute |
| - Lines 145 and 149: Not necessary to specify that articles were screened or reviewed by the coauthors. | - Removed language about co-authors’ specific responsibilities in screening, rephrased for conciseness and clarity |
| - Lines 148-149: Item “d” specifies pathogens, but does this include toxins? Please clarify. | - Good question from the reviewers. The inclusion criterion does include toxins and the search encompassed all agents which result in the diseases of interest listed in Table 1. However, the language in this line was rephrased to clarify that both types of disease agents were included. |
| - Line 158-162: Syntax and organization of list is distracting, please re-word. | - Revised the language of these lines as suggested |
| - Consider moving Figure 1 to methods section. | - The authors value this suggestion but in keeping with the format of recently published Plos One systematic reviews, we would prefer to keep Figure 1 in the Results section of the manuscript |
| **Results**   - Figure 1: Consider specifying on figure or in legend why records were excluded during screening; information is included in the text but would be helpful in the figure. | - The authors included this information in Figure 1. It is located on the right side of the image within the box titled, “Full-Text Articles Excluded, With Reasons” |
| - Line 175: Suggest updating paragraph header to reflect the fact that both pathogens and toxins are discussed. | - The phrase “*and Toxins*” was added per the reviewer prompt |
| - Line 191: *K. brevis* isn’t a pathogen, it’s a toxin-producing organism. Please clarify. | - The phrase “*brevetoxins produced by*” was inserted |
| - Suggest including a map with colors to indicate where in the state the authors are reporting studies on various types of animal disease and what types of pathogens are there. This could help vets and public health officials understand where animal diseases of public health relevance have been reported. | - As suggested by the reviewer(s), a map figure (Figure 2) was created and was added to help demonstrate the geographical density of studies by animal type and disease category |
| - Suggest creating a table or figure with results broadly summarized/synthesized, reflecting text; for example, it would be helpful to see how many studies were associated with each disease agent, animal type, and location, rather than a list of studies. | - A new map figure was created to summarize the included studies by animal type, location and disease agent as recommended by the reviewer (Figure 2) - Additional table information was also added to show the prevalence data for the sampled animals, when available |
| **Discussion**   - Line 231: Add period after citations. | - This was corrected. Thank you. |
| - Line 296: K. brevis isn’t a toxin, it produces toxins (brevetoxins). | - Revised language throughout this paragraph to reflect that brevetoxins are produced by *K. brevis.* |
| - Line 312: Toxins are not pathogens, please revise text. | - Corrected the word “*infected*” to “*affected*” to reflect differences between pathogens and toxins - Revised the word “*pathogen*” to “*disease-causing agent*” to reflect differences between pathogens and toxins |
| - In general, the discussion is focused on recommendations to reduce risks associated with different types of animal-associated human disease agents, and broad descriptions of included study results. However, the systematic review was aimed at enumerating reports of animal-associated human disease agents, and it is not clear how the results of the review inform the key points in the discussion. Suggest that the authors revise the discussion with a focus on describing broad trends (animals, diseases, and locations) they found in their literature search, how this information could be utilized by the state and/or vets and public health personnel, and identifying opportunities for additional research. | - The authors recognize a limitation of this paper is that we may have unintentionally excluded discipline-specific journals that cover topics such as veterinary science and zoology, which were not indexed by the search databases we utilized. Our intention is to bridge the findings presented by animal health scientists for public health and veterinary collaboration in the future. For example, this review could be used by veterinarians to consider screening animals based on recommendations we have outlined here for captive and companion animals and for epidemiologists to add additional zoonotic risk factors to their case investigations for reportable human disease. - The authors have added text to further discuss One Health importance of sharing information across counties, particularly those that represent a gap in published works related to animal prevalence of the pathogens and toxins investigated by this review. Figure 2 will ideally also help to illustrate the dearth of studies that have been done across the state and invite new research into these regions. |
