# Supplementary material for: A systematic review of waterborne and water-related disease in animal populations of Florida from 1999–2019
Source: PLoS One. 2021 Jul 29;16(7):e0255025. doi: 10.1371/journal.pone.0255025 (PMC8321142; doi:10.1371/journal.pone.0255025)
Supplement: Attachment — Submitted filename: PLOS ONE rev 2.docx [file pone.0255025.s005.docx]

The authors of “A systematic review of waterborne and water-related disease in animal populations of Florida from 1999-2019” made several changes to improve the manuscript’s quality, clarity, and adherence to PLOS ONE’s guidelines for systematic reviews. The inclusion of the new map figure, table, and clarification of the terminology around toxins v. pathogens were especially appreciated. Specific comments and suggestions on the revised manuscript are noted below.

Abstract

- Line 44: Clarify that these are not necessarily the diseases that have been circulating in animals, but those that have been reported on.

Introduction

- Line 75: Include an example of a vector here; the author’s include examples of vectors below (line 80), but suggest moving this to line 75 where vectors are first mentioned.
- Paragraph beginning at line 78: Seems a bit repetitive with the preceding paragraph (definitions of toxins, pathogens, etc.). Suggest combining these two paragraphs to improve flow. It’s also not clear why toxins are mentioned in the preceding paragraph but not the one starting at line 78.
- Lines 86-88: Suggest also mentioning that some of these diseases are reportable because they’re there is a need to control for potential person-to-person transmission after zoonotic infection.
- Lines 94-99: Suggest rewording or adding a sentence that explicitly states that while you’re reporting on the number of studies, this does not necessarily correlate to risk or prevalence associated with a given disease agent.

Results

- The authors have made a number of revisions to clarify pathogens v. toxins in the manuscript. However, in lines 211 and 219 and in Table 2, K. brevis is seemingly referred to as a toxin. Suggest clarifying throughout that K. brevis is a toxin-producer and not a toxin or pathogen itself and differentiating toxins (like saxitoxin) from toxin-producers (like K. brevis). For example, the authors could change “frequently examined pathogens and toxins” to “frequently examined pathogens and toxin-producers” in line 211.

Discussion

- Suggest adding subheadings.
- Line 261: Suggest changing “found” to “reported”.
- Line 263: Use abbreviation E. coli here since the full taxonomic name is listed in line 261.
- Line 264: Suggest using more precise language to describe E. coli; E. coli is a species with subtypes (strains) that are pathogenic.
- Line 266: FDOH abbreviation not previously defined.
- Line 269: If pathotype wasn’t described, how did the study authors use E. coli? Perhaps as a fecal indicator? This wouldn’t necessarily fit in with your description of E. coli as a pathogen, but any detectable E. coli might be problematic since it is used as fecal indicator and feces in general carry a wide array of disease agents (Salmonella, Campy, norovirus, etc.). The authors seem to be hinting at this later in the same paragraph, but this should be clarified if E. coli is included as a specific discussion point.
- Line 271 to end of paragraph: Is this section intended to be specific for E. coli or all bacterial enteropathogens? Please clarify.
- Suggest adding additional detail to the discussion of the places where you found the most studies of various animals and diseases (line 435). The map shows some interesting geographic trends with regard to the types of animals and pathogens/toxins being looked at and the total number of studies from in various parts of the state. Is this distribution related ecology? Funding? University locations? Population size? What areas might benefit from additional research based on the results of this study?

Figures

- It may just be the pdf version created for reviewers, but both figures are poor resolution and difficult to read; suggest improving figure resolution.
