# Supplementary material for: A systematic review of waterborne and water-related disease in animal populations of Florida from 1999–2019
Source: PLoS One. 2021 Jul 29;16(7):e0255025. doi: 10.1371/journal.pone.0255025 (PMC8321142; doi:10.1371/journal.pone.0255025)
Supplement: Attachment — Submitted filename: PLOS ONE rev 2_June2021_Comments.docx [file pone.0255025.s006.docx]

**The authors of “A systematic review of waterborne and water-related disease in animal populations of Florida from 1999-2019” are very grateful for the reviewer who has continued to provide support and advice for improvement of the manuscript. We have attempted to address all concerns outlined below and will be very willing to return to the paper to continue editing as needed.**

**Reviewer comments and author responses below:**

Abstract

- Line 44: Clarify that these are not necessarily the diseases that have been circulating in animals, but those that have been reported on.
  - ***Author Response: Sentence changed to: Findings from this review elucidate the water-related disease-causing pathogens and toxins which have been reported within animal populations in recent Florida history.***

Introduction

- Line 75: Include an example of a vector here; the author’s include examples of vectors below (line 80), but suggest moving this to line 75 where vectors are first mentioned.
  - ***Author Response: Vector example of mosquito was moved to the first mention of a water-related vector-borne disease (lines 78-79).***
- Paragraph beginning at line 78: Seems a bit repetitive with the preceding paragraph (definitions of toxins, pathogens, etc.). Suggest combining these two paragraphs to improve flow. It’s also not clear why toxins are mentioned in the preceding paragraph but not the one starting at line 78.
  - ***Author Response: The authors agree and have combined the paragraphs to avoid repetition as suggested by the reviewer.***
- Lines 86-88: Suggest also mentioning that some of these diseases are reportable because they’re there is a need to control for potential person-to-person transmission after zoonotic infection.
  - ***Author Response: The authors appreciate this suggestion and have incorporated it into the revised passage at lines 85-86.***
- Lines 94-99: Suggest rewording or adding a sentence that explicitly states that while you’re reporting on the number of studies, this does not necessarily correlate to risk or prevalence associated with a given disease agent.
  - ***Author Response: Excellent note. The authors have reworked the study objective language in lines 115-118 to state, “Although the included studies do not correlate directly to human disease risk or prevalence, understanding which water-related pathogens and toxins impact Florida’s animals in recent years can illuminate ways to prevent transmission in both populations”.***

Results

- The authors have made a number of revisions to clarify pathogens v. toxins in the manuscript. However, in lines 211 and 219 and in Table 2, K. brevis is seemingly referred to as a toxin. Suggest clarifying throughout that K. brevis is a toxin-producer and not a toxin or pathogen itself and differentiating toxins (like saxitoxin) from toxin-producers (like K. brevis). For example, the authors could change “frequently examined pathogens and toxins” to “frequently examined pathogens and toxin-producers” in line 211.
  - ***Author Response: The authors are grateful for this note and have added language throughout the document where K. brevis is addressed to indicate it is a toxin-producer and not a toxin itself, including in the column heading of Table 2.***

Discussion

- Suggest adding subheadings.
  - ***Author Response: Subheadings have been added to the discussion section.***
- Line 261: Suggest changing “found” to “reported”.
  - ***Author Response: Wording changed to reported (line 286).***
- Line 263: Use abbreviation E. coli here since the full taxonomic name is listed in line 261.
  - ***Author Response: Changed to the abbreviation (line 288)***
- Line 264: Suggest using more precise language to describe E. coli; E. coli is a species with subtypes (strains) that are pathogenic.
  - ***Author Response: Passaged changed to now read, “While E. coli is a broad species of bacterial subtypes….” per reviewer guidance (lines 288-289).***
- Line 266: FDOH abbreviation not previously defined.
  - ***Author Response: The abbreviation FDOH was added in brackets to the definition Florida Department of Health in the results section (line 145).***
- Line 269: If pathotype wasn’t described, how did the study authors use E. coli? Perhaps as a fecal indicator? This wouldn’t necessarily fit in with your description of E. coli as a pathogen, but any detectable E. coli might be problematic since it is used as fecal indicator and feces in general carry a wide array of disease agents (Salmonella, Campy, norovirus, etc.). The authors seem to be hinting at this later in the same paragraph, but this should be clarified if E. coli is included as a specific discussion point.
  - ***Author Response: The authors appreciate this point and the guidance provided by the reviewer. Additional clarification was provided in the discussion section (lines 294-296) and a note was added to the limitations (lines 499-501)***
- Line 271 to end of paragraph: Is this section intended to be specific for E. coli or all bacterial enteropathogens? Please clarify.
  - ***Author Response: Sentence changed to read, “The indiscriminate water-focused fecal shedding of zoonotic pathogens, such as pathogenic E. coli, from marine mammals is a point of concern since the state thrives on water-based tourism and recreation” (lines 303-304)***
- Suggest adding additional detail to the discussion of the places where you found the most studies of various animals and diseases (line 435). The map shows some interesting geographic trends with regard to the types of animals and pathogens/toxins being looked at and the total number of studies from in various parts of the state. Is this distribution related ecology? Funding? University locations? Population size? What areas might benefit from additional research based on the results of this study?
  - ***Author Response: Excellent question. Additional information was provided to the discussion section at lines 471-477 to outline a possible reason for the abundance of studies in some counties and a dearth of studies in others. However, without contact information provided within the articles, the site and source of specimens is a guess just based on the location of the sampling. The authors believe that specific contact information regarding study site and research teams is purposeful in the articles to avoid any public concern or confusion that could harm tourism or interest in the region.***

Figures

- It may just be the pdf version created for reviewers, but both figures are poor resolution and difficult to read; suggest improving figure resolution.
  - ***Author Response: The figures have been uploaded to PACE to help with clarity issues and the new versions will be submitted with these edits. Thank you.***
